# Quality and Reliability of Adolescent Sexuality Education on Chinese Video Platforms: Sentiment-Topic Analysis and Cross-Sectional Study

**DOI:** 10.2196/77100

**Published:** 2025-09-05

**Authors:** Lan Wang, Xiantao Shu, Jianmei Huang, Weiqian Yan, Duo Zhao

**Affiliations:** 1 Department of Obstetrics and Gynecology Huai'an 82 hospital Huai'an China; 2 Medical School of Yangtze University Jingzhou China; 3 Department of Orthopedics The First People's Hospital of Guannan: Lianyun Lianyungang China

**Keywords:** adolescent sexuality education, videos quality, Bilibili, TikTok, Kwai, Global Quality Score, GQS, modified DISCERN, mDISCERN, Patient Education Materials Assessment Tool-Audiovisual, PEMAT-A/V, information reliability, China, Spearman correlation analysis, stepwise regression analysis, topic modeling, sentiment analysis

## Abstract

**Background:**

Adolescence is a critical period for lifelong health, which makes access to accurate and comprehensive sexuality education essential. As video platforms become a primary source of information for adolescents, the quality of their content significantly impacts their physical and mental health.

**Objective:**

This study aimed to evaluate the quality, reliability, understandability, and actionability of adolescent sexuality education videos on major Chinese platforms (Bilibili, TikTok or Douyin, and Kwai), analyze associated user comment sentiment and topics, identify predictors of quality and reliability, and provide recommendations.

**Methods:**

A cross-sectional analysis was conducted (April 2025) on the top 100 comprehensively ranked comprehensive sexuality education videos (N=300 total) retrieved from each platform using the keyword 青春期性教育 (“adolescent sexuality education”). Videos were assessed using the Global Quality Score, modified DISCERN, and Patient Education Materials Assessment Tool (PEMAT-U/A), with interrater reliability assessed via Cohen κ. A corpus of over 49,000 user comments underwent sentiment analysis (fine-tuned RoBERTa) and topic modeling (BERTopic, yielding 29 topics grouped into 6 themes). Statistical analyses included Kruskal-Wallis *H* tests, Spearman correlations, and stepwise linear regressions (SPSS [version 27.0]; *P*<.05).

**Results:**

Video quality and reliability were moderate on Bilibili and TikTok but generally poor on Kwai. Content from verified sources (physicians, educators, and institutional media) demonstrated superior quality and stability compared to highly variable content from individual media (the predominant source type, especially on Kwai; 87/100, 87%). Paradoxically, Kwai exhibited the highest user engagement despite the lowest quality scores. Understandability (PEMAT-U) was consistently the strongest positive predictor for both quality (Global Quality Score, final model adjusted *R*^2^=0.383, β=0.485) and reliability (modified DISCERN, final model adjusted *R*^2^=0.209, β=0.319). Actionability (PEMAT-A) and video duration were also significant positive predictors. Understandability scores (PEMAT-U) were generally high (approximately 69%), while actionability scores (PEMAT-A) were moderate to low (33%-50%). Sentiment analysis revealed that comments were predominantly neutral (35,372/49,680, 71.2%), with negative comments (9141/49,680, 18.4%) significantly outweighing positive ones (5167/49,680, 10.4%). Key discussion themes identified included sources of knowledge acquisition, sexual safety and prevention, physiology, and sexual health and practices.

**Conclusions:**

While online video platforms offer accessible channels for adolescent sexuality education in China, the current content is often of moderate-to-poor quality, with questionable reliability and limited actionability. Understandability is paramount, but high engagement does not necessarily correlate with high quality or reliability, potentially amplifying misinformation. To effectively empower youth, critical steps include enhancing content quality by adhering to evidence-based frameworks like the International Technical Guidance on Sexuality Education; strengthening platform accountability through improved verification and algorithms; and promoting user media literacy. These measures aim to foster a healthier and more equitable future for Chinese adolescents, helping to achieve goals related to sexually transmitted infections and pregnancy prevention and promoting more open societal attitudes toward sexuality.

## Introduction

### Background

Adolescence represents a critical developmental window for understanding sexuality, health, and relationships, shaped by complex sociobiological interactions [1]. Comprehensive sexuality education (CSE), grounded in human rights and gender equality principles as outlined by the International Technical Guidance on Sexuality Education (ITGSE) framework [2], is vital for equipping young people for safe, fulfilling lives [3]. Increasingly, adolescents turn to readily accessible online sources, particularly video platforms, for sexual health information [4,5]. This digital shift presents both significant opportunities for wide-reaching health communication and considerable challenges regarding content quality, accuracy, and impact.

Despite the potential of digital platforms for advancing sustainable development goals (SDGs) related to health (SDG 3), education (SDG 4), and gender equality (SDG 5), the quality and reliability of online CSE content are major global concerns. Consistent with ITGSE warnings, adolescents navigating the abundant digital landscape are susceptible to misinformation and biased perspectives, often amplified by unverified content creators (lacking platform-validated authentication of declared qualifications or professional status). Current digital content frequently lacks inclusivity, overlooking the specific needs and experiences of diverse youth, including lesbian, gay, bisexual, transgender, queer, two-spirit, quantum+ individuals and those with disabilities, groups who already face elevated risks of violence and barriers to Sexual and Reproductive Health information and care [6-8]. Furthermore, the typical short-video format, often prioritizing user engagement metrics over pedagogical substance, may fail to deliver the depth and progressive learning structure advocated by CSE frameworks such as ITGSE. The widespread availability of potentially harmful content, such as pornography, poses additional risks to healthy adolescent development, while the circulation of inaccurate reproductive health information can negatively impact health outcomes and trust in health care systems [9].

These challenges are particularly pronounced in China. The country boasts of the world’s largest adolescent population [10]; however, it faces significant deficiencies in formal sex education infrastructure, including a scarcity of trained educators and age-appropriate materials. This gap is reflected in the low levels of sexual and reproductive health knowledge among young people [10,11]. This knowledge deficit is not trivial; it directly correlates with a range of severe public health issues, such as increased risks of adolescent pregnancies, the spread of sexually transmitted infections (STIs) and HIV, and heightened vulnerability to sexual violence. Specific vulnerable groups, such as rural left-behind children, encounter unique difficulties [12], and disparities exist between urban and rural adolescents in relevant knowledge [4], making them more susceptible to these negative health outcomes. The prevalent parental reluctance to openly discuss sexual topics further exacerbates these problems [13], leaving adolescents without necessary guidance and support when confronting sexual health challenges. In this context, online video platforms, such as Bilibili, Douyin, and Kuaishou have become primary information channels for young people [4]. While existing research has assessed the quality of online health videos for various other medical conditions [14-21], a systematic evaluation of adolescent CSE video quality, reliability, and user feedback on these major platforms—where millions of adolescents seek information—is conspicuously lacking, despite its clear public health significance.

### This Study

Addressing this critical knowledge gap, this study aimed to comprehensively evaluate adolescent sexuality education video content disseminated via Bilibili, TikTok, and Kwai in China. Grounded in the ITGSE framework’s tripartite foundation (knowledge, attitudes, and skills) [2], we assessed video quality (Global Quality Score [GQS]), reliability (mDISCERN), understandability (Patient Education Materials Assessment Tool-Understandability [PEMAT-U]), and actionability (Patient Education Materials Assessment Tool-Actionability [PEMAT-A]) using validated instruments and expert raters. Departing from solely content-focused analyses, we innovatively integrated computational methods by performing sentiment analysis and topic modeling (BERTopic) on a large corpus of associated user comments (approximately 49,000) to capture audience reception and discourse. By synthesizing evidence from video assessments, user feedback analysis, and platform comparisons, this research sought to identify current strengths, critical shortcomings, and strategic opportunities for enhancing digital CSE approaches to effectively empower adolescents.

## Methods

### Data Collection and Screening

**Figure 1:**
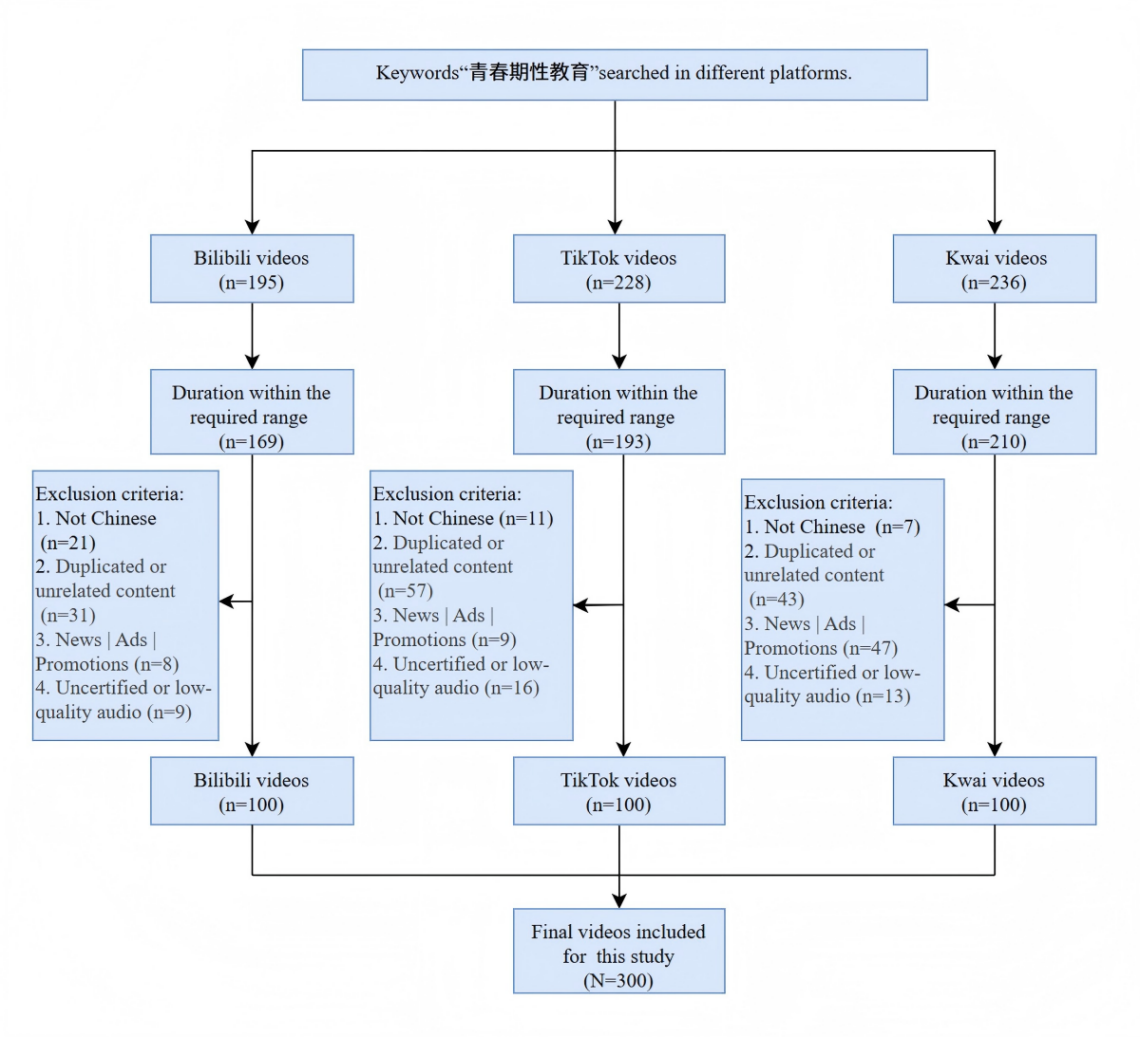
Flowchart for searching adolescent sex education videos.

This study used a cross-sectional approach. Between April 1 and April 4, 2025, new accounts were created on Bilibili, TikTok, and Kwai to perform video searches using the precise Chinese term 青春期性教育 (*qīngchūnqī xìngjiàoyù*, “adolescent sexuality education”). For each platform, the 100 top-ranked videos according to the platform’s internal algorithm were initially gathered for evaluation (Figure 1). Videos were included if they met three conditions: (1) the content addressed adolescent sexuality education, (2) the language used was Mandarin Chinese, and (3) the video length fell between 20 seconds and 20 minutes. Content was excluded based on several factors: (1) irrelevance to the research topic, (2) duplication or high similarity to other selected videos, (3) function as commercial advertising, (4) presentation as news reporting, (5) use of languages other than Chinese, (6) absence of audio or severely poor sound quality, and (7) uploads originating from unverified platform users. Application of these criteria resulted in a final sample of 300 videos, with an equal count of 100 videos derived from each of the 3 platforms (Bilibili, TikTok, and Kwai). The video content was independently reviewed and verified by 2 researchers. The subsequent analysis was intentionally restricted to these top 100 videos per platform, a decision informed by existing methodological studies, which indicate that videos ranking lower typically add minimal value and do not substantially change overall findings in comparable platform content research [22,23].

### Data Collection Content

Key characteristics were documented for every video incorporated in the final sample. These systematically recorded parameters included the source or affiliation of the uploader, the video’s runtime (duration), and several indicators of user interaction, such as counts for likes, favorites (also termed collections), shares, comments, along with the uploader’s total number of followers. This dataset was organized and stored using Microsoft Excel software (Multimedia Appendix 1).

To enable systematic analysis, a video classification scheme was implemented. Each platform was assigned a numerical identifier (Bilibili=1, TikTok=2, and Kwai=3). On the basis of the principal subject matter, videos were sorted into five thematic content groups: (1) knowledge (covering aspects such as the evolution and current landscape of adolescent sexuality education, its significance, physiological and psychological development during puberty, educational policies, and contraception use), (2) prevent sexual assault, (3) sexual behavior, (4) AIDS, and (5) other sexually transmitted diseases (STDs). Furthermore, the originators of the content were classified into four source types: (1) individual media (persons operating independently), (2) institutional media (entities, such as official bodies or established media companies), (3) physician (inclusive of psychologists, practitioners of modern medicine, and practitioners of traditional Chinese medicine), and (4) educator.

Web scraping techniques yielded an initial dataset of 86,647 user comments from 3 Chinese social media platforms: Bilibili, Douyin (TikTok), and Kwai. These raw data underwent a standardized cleaning protocol involving normalization, noise removal, and elimination of duplicate entries, resulting in a final analytic corpus containing roughly 49,000 comments. Concurrently, for the purpose of supervised model development, a distinct subset originating from 1999 raw comments was curated. After applying identical preprocessing steps, this subset was partitioned using stratified sampling into training (1699/1999, 85%) and validation (300/1999, 15%) datasets.

### Quality Assessment Tools

#### GQS

Ranging from 1 to 5 points, with higher scores indicating superior quality [24], this metric evaluates accuracy, authority, completeness, and educational value.

#### Modified DISCERN Tool

Comprising 5 questions adapted from the original DISCERN (Decision Instrument for Screening Consumer Ehealth Resources aNd tools) tool, it is used to assess the reliability of health information [17], evaluating clarity, relevance, traceability, scientific basis, and impartiality. Each dimension is scored 1 point for “yes” and 0 points for “no” (Multimedia Appendix 2).

#### PEMAT

The PEMAT tool for audiovisual materials (PEMAT-A/V) with an automated scoring form assesses the understandability and actionability of videos [25].

### Statistical Analysis

All statistical analyses were performed using SPSS software (version 27.0; IBM Corp). Key variables were summarized descriptively, presenting medians alongside IQRs. To examine variations between platforms concerning several metrics (such as video duration, likes, and collections), the nonparametric Kruskal-Wallis *H* test was applied. Relationships between video quality assessments and user engagement metrics were evaluated via Spearman rank-order correlation. Furthermore, predictive models were constructed through the application of stepwise linear regression techniques. Drawing figures with R (version 4.4.2; R Foundation for Statistical Computing), the degree of agreement between the 2 expert raters for quality scores was quantified using Cohen κ statistic. For all inferential statistical tests conducted, the threshold for significance was established at *P*<.05.

For classifying comment sentiment polarity, we used the hfl/chinese-roberta-wwm-ext model [26] after fine-tuning. Implementation relied on the transformers library [27], with training conducted over 3 epochs using a 3e-5 learning rate. The optimal model iteration was chosen based on maximizing the weighted *F*_1_-score obtained on the designated validation dataset. To ensure the reliability and consistency of performance findings, the complete training and assessment cycle was executed 10 times independently, each initiated with a unique random seed (eg, 42, 13, 95, etc). Final performance measures were subsequently presented as means accompanied by their SDs.

Latent thematic structures within the comment data were identified using the BERTopic framework [28], which operated on document vector representations precalculated via the text2vec-base-chinese model [29]. This process incorporated dimensionality reduction via uniform manifold approximation and projection [30], set with parameters n_components=10 and min_dist=0.0, succeeded by density-based clustering using Hierarchical Density-Based Spatial Clustering of Applications with Noise (HDBSCAN) [31], requiring a minimum cluster size of 180 comments. The resulting topic clusters were characterized by representative keywords derived from a class-based term frequency-inverse document frequency vectorization strategy, incorporating both single words and 2-word phrases (1-2 n-grams). Implementation of these core methodologies relied upon the Python programming language and its related ecosystem for scientific computation.

### Ethical Considerations

This study strictly adhered to research ethics guidelines. This study constitutes a secondary analysis of publicly available data. All data were sourced from openly accessible videos on Bilibili, TikTok, and Kwai platforms. The research did not involve any clinical records, human biological samples, or animal experimentation data. The study exclusively used publicly visible video content from these platforms. No personally identifiable information or private user data were collected, processed, or analyzed. All data were anonymized to prevent identification of specific individuals. As this study did not involve direct contact with or intervention among platform users, and only used publicly available anonymized data, it was exempt from ethics review board approval in accordance with international ethical standards and institutional review board regulations. Since the research data were entirely obtained from public platforms and did not compromise user privacy, obtaining participant consent was not required.

## Results

### Videos Characteristics

A summary of the general features of the analyzed videos can be found in Tables 1 and 2. The nonparametric Kruskal-Wallis *H* test served to identify overall variations among Bilibili, TikTok, and Kwai regarding several key indicators (such as duration, likes, collections, shares, and comments). Findings from this test indicated that the platforms differed significantly concerning both user engagement measures and the assessed scores for content quality and reliability. Therefore, the Dunn test was used for subsequent pairwise comparisons between the platforms to pinpoint specific differences. To control for the increased probability of type I errors arising from multiple comparisons, adjusted *P* values were calculated. Table 3 provides a detailed account of these post hoc comparison results.

As illustrated in Tables 4 and 5, which detail source and content distributions along with video features, individual media emerged as the most frequent source type overall. Creators operating independently contributed nearly two-thirds (199/300, 66.3%) of all videos analyzed across the Bilibili, TikTok, and Kwai platforms. This dominance by individual creators was especially pronounced on Kwai, where they accounted for 87% (87/100) of the content. Ranking significantly behind individual contributions were videos sourced from physicians (55/300, 18.3%) and educators (29/300, 9.7%), while institutional media provided the fewest videos (17/300, 5.7%). Distinct differences were observed between platforms regarding source prevalence. TikTok featured the highest concentration of content from physicians (33/100, 33%). Strikingly, the Kwai platform sample contained no videos produced by users identified as educators.

An examination of content themes revealed that “knowledge” concerning adolescent sexuality education was the most commonly encountered category; this theme accounted for 74% (74/100), 65% (65/100), and 75% (75/100) of the videos sampled from Bilibili, TikTok, and Kwai, respectively. Materials discussing “sexual behavior” also represented a substantial area of focus in the analyzed content.

Post hoc analyses revealed substantial differences between Bilibili and TikTok; specifically, the videos by Bilibili showed significantly elevated medians for runtime of 262 seconds (IQR 159-497), likes of 2345 (IQR 477-9354), collections of 1251 (IQR 183-3531), shares of 397 (IQR 72-1096), and comments of 189 (IQR 36-686) when contrasted with TikTok’s content (all *P*<.001). When comparing Bilibili and Kwai, videos on the Bilibili platform maintained a significantly longer median duration (*P*<.001), although they garnered significantly fewer shares compared to those on the Kwai platform (*P*<.05). A comparison between TikTok and Kwai indicated that TikTok content received significantly less user engagement in terms of median likes of 121 (IQR 30-1066), collections of 40 (IQR 8-317), shares of 40 (IQR 9-500), and comments of 8 (IQR 2-51), all *P*<.001. However, the median video duration for these 2 platforms did not differ statistically (adjusted *P*=.542).

Assessments of quality and reliability revealed platform-specific characteristics. Bilibili videos registered a median GQS of 3 (IQR 3-4) alongside a median modified DISCERN (mDISCERN) score of 3 (IQR 2-3). In comparison, TikTok videos presented median scores of 3 (IQR 2-3) for GQS and 2 (IQR 2-3) for mDISCERN. The GQS scores for Bilibili and TikTok did not exhibit a statistically significant difference (adjusted *P*=.052), implying similar overall quality levels between these 2 platforms. However, when compared against Kwai, with a median GQS of 2 (IQR 2-3) and median mDISCERN of 2 (IQR 2-2), Bilibili content achieved significantly superior scores on both GQS and mDISCERN (both *P*<.001). A significant disparity in GQS was also identified between TikTok and Kwai (*P*<.001), but their reliability scores (mDISCERN) were statistically comparable (adjusted *P*=0.200). The consistency between the 2 independent assessors for the quality evaluations was measured via Cohen κ. Applying the benchmarks suggested by Landis and Koch [32] (κ>0.8: excellent; 0.6-0.8: substantial; 0.4-0.6: moderate; <0.4: poor), the obtained κ coefficients for GQS (κ=0.779) and mDISCERN (κ=0.786) both fall within the range indicating substantial interrater agreement.

Evaluation with the PEMAT-U indicated that video understandability was uniformly high across the studied platforms. Bilibili with a median of 69% (IQR 62%-77%), TikTok with a median of 69% (IQR 67%-77%), and Kwai with a median of 69% (IQR 63%-77%), all exhibited similar median scores with no statistically significant interplatform variation detected. In contrast, actionability (PEMAT-A) scores were considerably lower, ranging from moderate for Bilibili with a median of 50% (IQR 0%-67%) to low for TikTok with a median of 33% (IQR 33%-67%) and Kwai with a median of 33% (IQR 33%-59%), although these platform differences also failed to reach statistical significance. Taken together, these results indicate that while most videos were readily understandable, they generally lacked sufficient elements to empower viewers toward practical application or health-related decision-making, revealing a substantial need for improvement in content actionability.

**Table 1:** Characteristics of the videos on different platforms.

Variable	Bilibili (n=100), median (IQR)	TikTok (n=100), median (IQR)	Kwai (n=100), median (IQR)
Duration (s)	262 (159-497)	100 (63-179)	93 (55-160)
Likes	2345 (477-9354)	121 (30-1066)	7646 (375-39,400)
Collections	1251 (183-3531)	40 (8-317)	1011 (92-4402)
Shares	397 (72-1096)	40 (9-500)	960 (138-4206)
Comments	189 (36-686)	8 (2-51)	270 (15-3098)
Followers	43,000 (5258-226,250)	7272 (1356-127,750)	129,500 (25,000-793,250)
GQS^a^	3 (3-4)	3 (2-3)	2 (2-3)
mDIS^b^	3 (2-3)	2 (2-3)	2 (2-2)
PEMAT-U^c^ (%)	69 (62-77)	69 (67-77)	69 (63-77)
PEMAT-A^d^ (%)	50 (0-67)	33 (33-67)	33 (33-59)

^a^GQS: Global Quality Score.

^b^mDIS: modified DISCERN.

^c^PEMAT-U: Patient Education Materials Assessment Tool-Understandability.

^d^PEMAT-A: Patient Education Materials Assessment Tool-Accountability.

**Table 2:** Kruskal-Wallis H test across different platforms.

	Kruskal-Wallis *H* test (*df*)	Asymptotic significance^a^
Duration (s)	72.911 (2)	<.001
Likes	58.326 (2)	<.001
Collections	58.529 (2)	<.001
Shares	44.959 (2)	<.001
Comments	58.046 (2)	<.001
Followers	19.511 (2)	<.001
GQS^b^	41.343 (2)	<.001
mDIS^c^	20.672 (2)	<.001
PEMAT-U^d^	0.939 (2)	0.625
PEMAT-A^e^	2.159 (2)	0.34

^a^*P* values rounded to 3 decimal places. Values below .001 are reported as *P*<.001.

^b^GQS: Global Quality Score.

^c^mDIS: modified DISCERN.

^d^PEMAT-U: Patient Education Materials Assessment Tool-Understandability.

^e^PEMAT-A: Patient Education Materials Assessment Tool-Accountability.

**Table 3:** Dunn test across different platforms.

Comparison	1^a^-2^b^ (Z)	1-2 (unadjusted *P* value)^c^	1-2 (adjusted *P* value)	1-3^d^ (Z)	1-3 (unadjusted *P* value)	1-3 (adjusted *P* value)	2-3 (Z)	2-3 (unadjusted *P* value)	2 - 3 (adjusted *P* value)
Duration (s)	6.634	<.001	<.001	7.973	< .001	<.001	1.338	.181	.542
Likes	5.879	<.001	<.001	–1.282	.2	.6	–7.161	<.001	<.001
Collections	6.919	<.001	<.001	0.633	.527	.99	–6.286	<.001	<.001
Shares	4.057	<.001	<.001	–2.595	.009	.028	–6.652	<.001	<.001
Comments	6.178	<.001	<.001	–0.771	.441	.99	–6.95	<.001	<.001
Followers	2.101	.036	.107	–2.315	.0206	.0619	–4.415	<.001	<.001
GQS^e^	2.379	.017	.052	6.363	<.001	<.001	3.984	<.001	<.001
mDIS^f^	2.685	.007	.022	4.52	<.001	<.001	1.835	.067	.2
PEMAT-U^g^	0.613	.54	.99	0.957	.339	.99	0.344	.731	.99
PEMAT-A^h^	–0.999	.318	.955	0.435	.664	.99	1.433	.152	.456

^a^Bilibili.

^b^TikTok.

^c^Data are reported to 3 decimal places. *P* values less than 0.001 are indicated as *P*<001.

^d^Kwai.

^e^GQS: Global Quality Score.

^f^mDIS: modified DISCERN.

^g^PEMAT-U: Patient Education Materials Assessment Tool-Understandability.

^h^PEMAT-A: Patient Education Materials Assessment Tool-Accountability.

**Table 4:** Characteristics of the videos across sources.

Variables	Individual media (n=199)	Institutional media (n=17)	Physician (n=55)	Educator (n=29)
Duration (s), median (IQR)	136 (74-246)	210 (103-573)	90 (50-162)	199 (110-258)
Likes, median (IQR)	1803 (142-16,000)	286 (61-12,341)	458 (38-2359)	2328 (427-5574)
Collections, median (IQR)	532 (58-3313)	92 (11-2568)	200 (12-685)	825 (60-2535)
Shares, median (IQR)	435 (44-1877)	215 (24-5658)	147 (13-853)	374 (35-1341)
Comments, median (IQR)	89 (8-895)	18 (2-241)	17 (2-114)	175 (15-600)
Followers, median (IQR)	41,000 (1802-304,000)	94,000 (2428-825,500)	34,000 (2429-233,000)	58,000 (4997-110,500)
GQS^a^, median (IQR)	3 (2-3)	3 (3-4)	3 (3-4)	4 (3-4)
mDIS^b^, median (IQR)	2 (2-3)	3 (2-3)	2 (2-3)	3 (2-3)
PEMAT-U^c^ (%), median (IQR)	69 (62-77)	77 (69-85)	69 (67-77)	69 (69-81)
PEMAT-A^d^ (%), median (IQR)	33 (0-50)	67 (33-67)	50 (33-67)	50 (13-67)

^a^GQS: Global Quality Score.

^b^mDIS: modified DISCERN.

^c^PEMAT-U: Patient Education Materials Assessment Tool-Understandability.

^d^PEMAT-A: Patient Education Materials Assessment Tool-Accountability.

**Table 5:** Percentage distribution of adolescent sex education videos by source and content on different platforms.

Variable and subcategories		Bilibili, %	TikTok, %	Kwai, %
**Source**				
	Individual media	65	47	87
	Institutional media	6	8	3
	Physician	12	33	10
	Educator	17	12	0
**Content**				
	Knowledge	74	65	75
	Prevent sexual assault	7	1	3
	Sexual behavior	14	21	18
	AIDS	3	9	3
	Sexually transmitted disease	2	4	1

### Video Quality and Reliability Assessments

**Figure 2:**
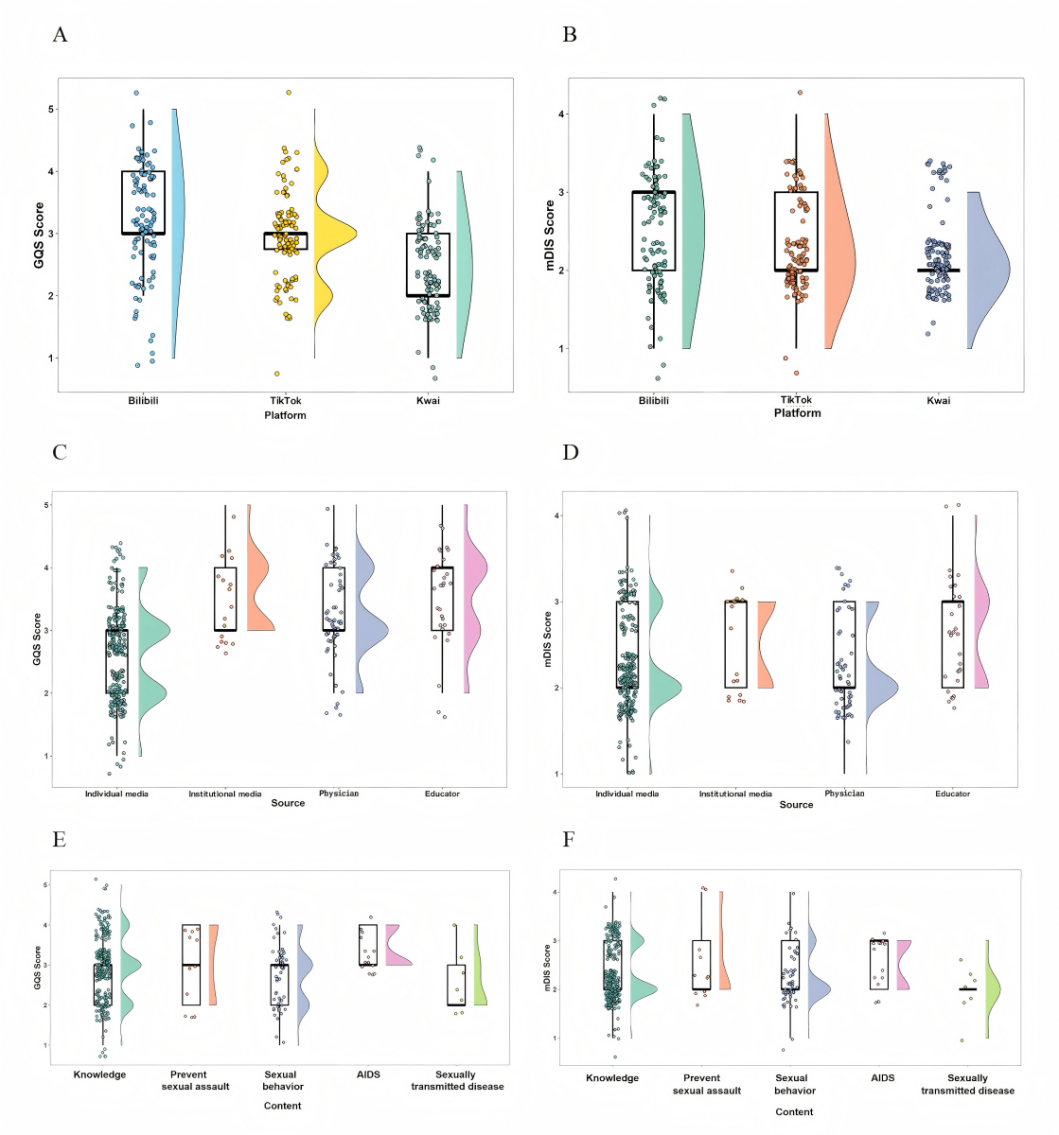
Global Quality Score (GQS) and modified DISCERN (mDISCERN) scores. (A) Comparison of GQS across different platforms. (B) Comparison of mDISCERN scores across different platforms. (C) GQS scores across different video sources. (D) mDISCERN scores across different video sources. (E) GQS scores across different content categories. (F) mDISCERN scores across different content categories..

Platform comparisons highlighted differing quality profiles based on GQS assessments (Figure 2A). Content on Bilibili frequently scored between 3 and 4, denoting relatively high quality. TikTok’s videos typically clustered around a score of 3, representing moderate quality. In contrast, Kwai content primarily ranged between 2 and 3, signifying comparatively lower quality. Reliability evaluations using mDISCERN (Figure 2B indicated moderate reliability for both Bilibili (scores concentrated between 2 and 3) and TikTok (median score of 2). However, Kwai presented a pattern skewed toward lower mDISCERN scores, suggesting inferior reliability overall. Synthesizing these results, while the overall quality difference between Bilibili and TikTok approached but did not reach statistical significance based on previous tests (adjusted *P*=0.052), the score distribution profile by Bilibili suggested stronger performance. Furthermore, Bilibili consistently demonstrated superior reliability compared to the other platforms. Kwai emerged as the platform with the lowest performance regarding both content quality and reliability metrics.

Examining scores based on the source of the video (Figure 2C and D), materials produced by institutional media, physicians, and educators typically clustered at the upper end of the GQS, signifying more dependable and higher-quality content. In contrast, while videos from individual media registered a median GQS of 3 (IQR 2-3) (Figure 2C), their distribution was notably bimodal, pointing to substantial variation in quality standards among these independent creators. With respect to reliability as measured by mDISCERN (Figure 2D), both institutional media and educators attained a median score of 3, outperforming the median of 2 recorded for the remaining source types.

When examining results based on content categorization (Figure 2E), videos covering knowledge, sexual assault prevention, sexual behavior, and AIDS typically achieved moderate GQS scores. In contrast, materials centered on STDs generally received evaluations corresponding to fair or lower-quality levels. Regarding reliability assessments (mDISCERN; Figure 2F), content specifically addressing AIDS distinguished itself by attaining relatively higher reliability scores compared to videos in the other thematic groups.

### Video Understandability and Actionability

**Figure 3:**
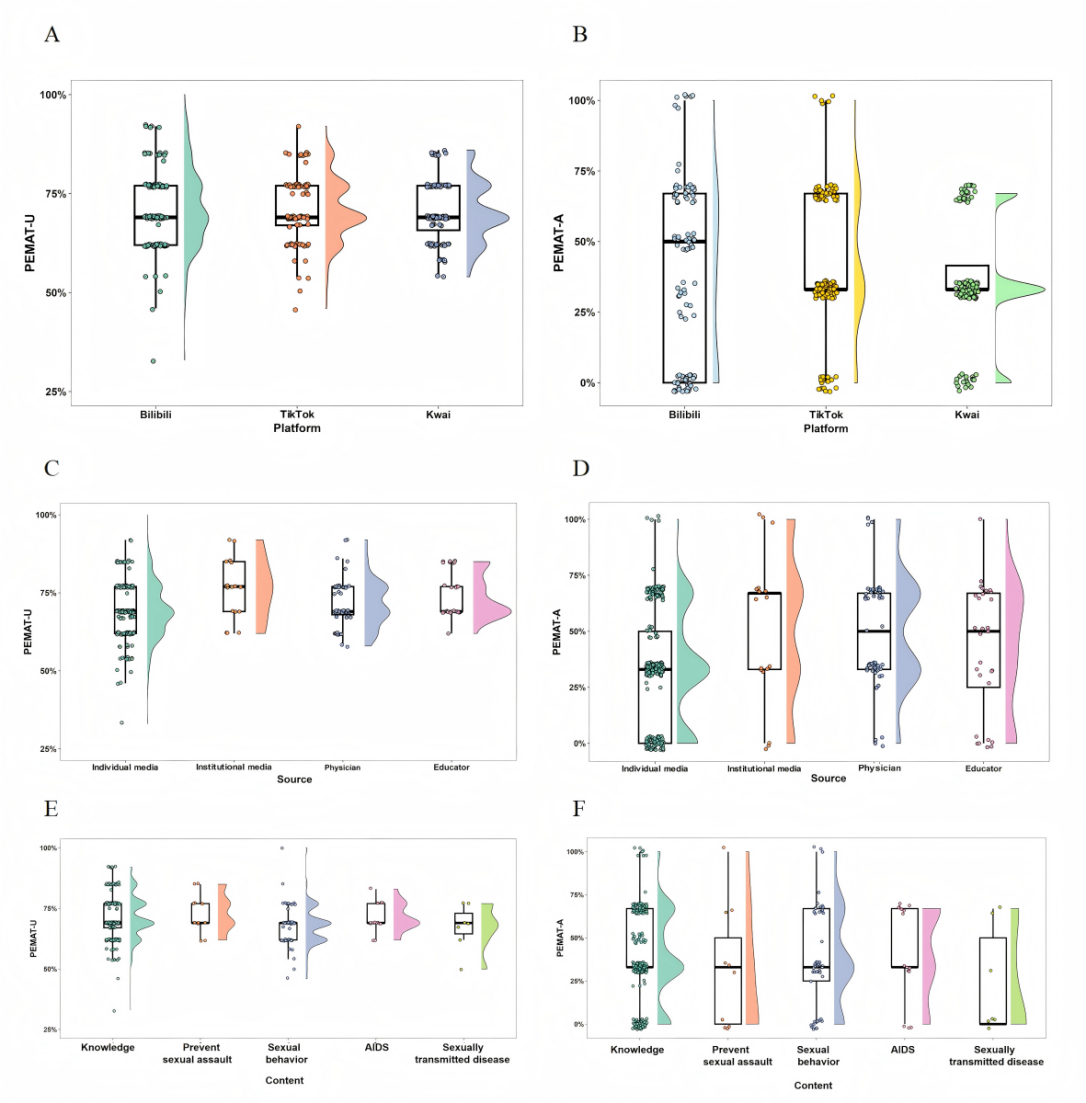
Comparative analysis of video understandability and actionability. (A) Patient Education Materials Assessment Tool-Understandability (PEMAT-U) scores of videos across platforms. (B) Patient Education Materials Assessment Tool-Accountability (PEMAT-A) scores of videos across platforms. (C) PEMAT-U scores of videos across sources. (D) PEMAT-A scores of videos across sources. (E) PEMAT-U scores of videos across content categories. (F) PEMAT-A scores of videos across content categories.

When PEMAT-U (understandability) scores were analyzed by platform (Figure 3A), a uniform median score of 69% was observed for Bilibili, TikTok, and Kwai. Despite identical medians, score distributions varied; TikTok’s scores clustered more tightly at the higher end, implying greater consistency in understandability, while Bilibili displayed a more polarized distribution, possibly due to its diverse range of content creators. Kwai, whose content primarily originates from individual media, often without professional expertise, registered moderate understandability scores but demonstrated notably deficient performance regarding actionability (Figure 3B).

Analysis based on video source categorization yielded divergent profiles for understandability and actionability (Figure 3C and D). Notably, institutional media outperformed other sources across both metrics. Conversely, while exhibiting considerable spread in understandability scores, content originating from individual media demonstrated particularly weak actionability; PEMAT-A scores for this group clustered at lower values, with a median of just 33%. However, materials created by physicians and educators showed less score dispersion, suggesting more consistent results in terms of both conveying comprehensible information and providing actionable guidance.

When analyzed by thematic area, most videos demonstrated proficient levels of understandability (PEMAT-U), as shown in Figure 3E. However, significant variability was observed within the “knowledge” content category; despite a median PEMAT-U of 69%, the scores ranged broadly from a minimum of 33% to nearly 100%, indicating inconsistent effectiveness in information presentation for this theme. Assessments of actionability (PEMAT-A; Figure 3F implied) revealed generally insufficient performance. Content focused on STDs was particularly inadequate in this regard, while materials covering other topics typically displayed only moderate actionability.

### Correlation and Stepwise Regression Analysis

**Figure 4:**
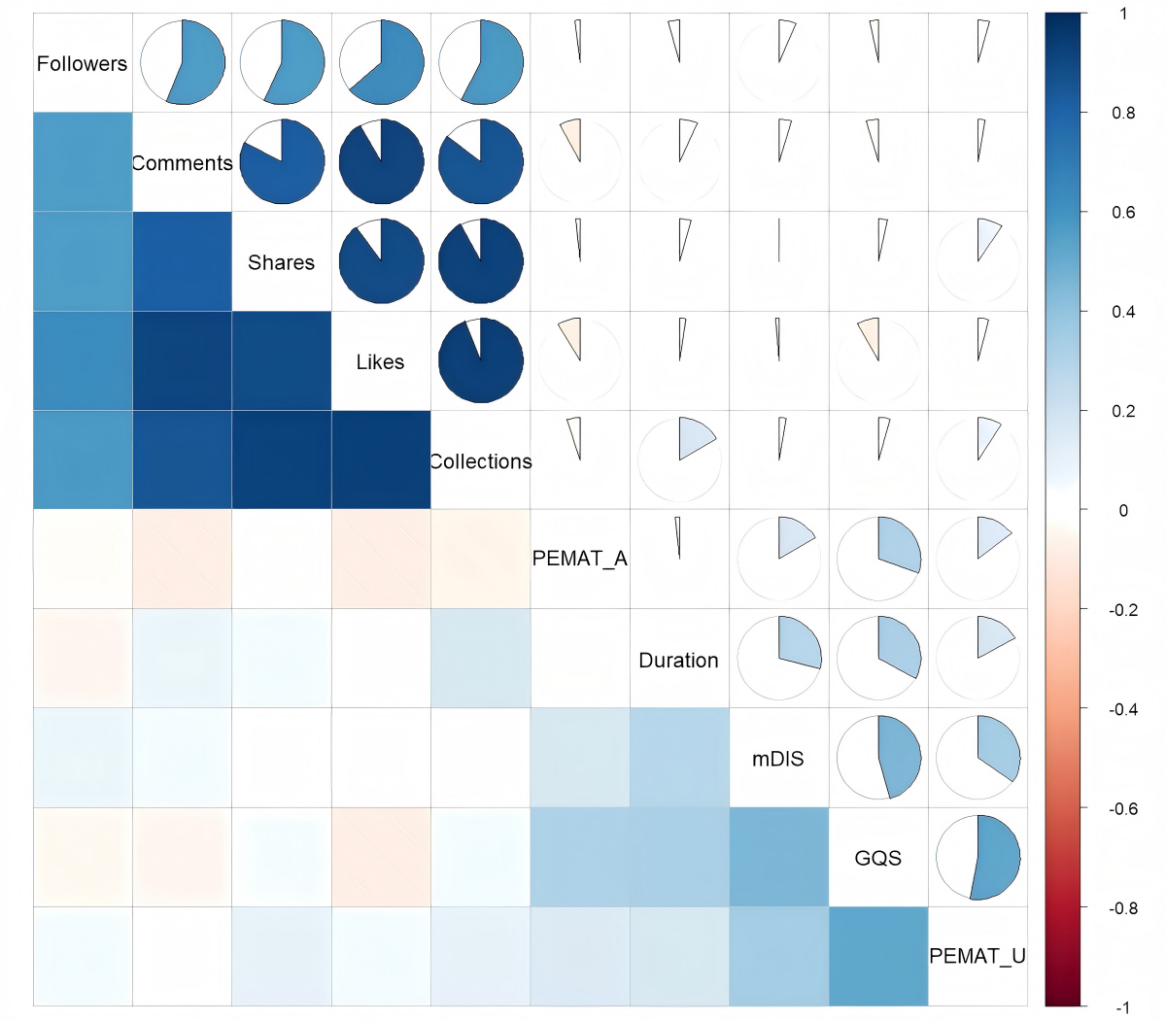
Correlation analysis. GQS: Global Quality Score; PEMAT-A: Patient Education Materials Assessment Tool-Accountability; PEMAT-U: Patient Education Materials Assessment Tool-Understandability;mDIS:modified DISCERN scores.

Further investigation into the interplay among variables involved Spearman rank correlation to evaluate connections between quality metrics (GQS and mDISCERN), user engagement levels, and video attributes; these associations are depicted in Figure 4. Results indicate a strong positive correlation between comments and likes, collections, and shares (*r=*0.83-0.94). A robust positive correlation was also observed between follower count and engagement metrics (*r=*0.56-0.64). Collectively, these indicators reflect content popularity and user engagement. The correlation between GQS and mDISCERN scores was *r*=0.46, with a significance level of *P*<.01, demonstrating a moderate positive relationship. Video duration showed correlations of *r*=0.29 with mDISCERN and *r*=0.33 with GQS.

Video understandability (PEMAT-U) exhibited a strong positive correlation with quality (GQS, *r*=0.53) and a moderate positive correlation with reliability (mDISCERN, *r*=0.34). Video actionability (PEMAT-A) showed a moderate positive correlation with GQS (*r*=0.30), but only weak positive correlations with mDISCERN and PEMAT-U (*r*=0.17 and *r*=0.15, respectively).

A stepwise linear regression procedure was implemented to ascertain key predictors for the GQS applied to adolescent sexuality education videos. This multistep process, detailed in Table 6, sequentially added variables maximizing explanatory contribution. The resultant final model (model 3) achieved statistical significance (*F*_3_=62.790, *P*<.001), accounting for 38.3% of the GQS variance (adjusted *R*^2^=0.383). Table 7 provides comprehensive details regarding the coefficients, significance levels, CIs, and collinearity checks for this concluding model. Findings confirmed positive and significant predictive roles for understandability (PEMAT-U: β=0.485, *t_296_*=10.317, *P*<.001, two-tailed), actionability (PEMAT-A: β=0.232, *t_296_*=5.040, *P*<.001, two-tailed), and video duration (β=0.186, *t_296_*=3.979, *P*<.001, two-tailed) in determining GQS. The significance of these positive associations was further supported by the 95% CIs for their unstandardized coefficients (B), which excluded 0: PEMAT-U (0.036-0.053), PEMAT-A (0.004-0.010), and duration (0.000-0.001). Moreover, diagnostic tests confirmed low multicollinearity among these predictors (tolerance>0.93, VIF<1.08), indicating stable coefficient estimations.

Stepwise linear regression was additionally used to ascertain significant determinants of video reliability, quantified via the mDISCERN score. The progression of model construction and specifics of the final coefficients are documented in Tables 8 and 9. The culminating 3-predictor model demonstrated overall statistical significance (*F*_3_=27.353, *P*<.001) and accounted for 20.9% of the variance observed in mDISCERN scores (adjusted *R*^2^=0.209). Table 8 details the final model parameters, confirming significant positive relationships between mDISCERN and all included predictors: understandability (PEMAT-U: β=0.319, *P*<.001; 95% CI for B [0.014-0.027]), duration (β=0.242, *P*<.001; 95% CI for B [0.000-0.001]), and actionability (PEMAT-A: β=0.126, *P*=.016; 95% CI for B [0.000-0.005]). On the basis of standardized coefficients, PEMAT-U exerted the most substantial relative influence, succeeded by duration, and lastly PEMAT-A. Low multicollinearity among these variables (all VIF<1.08) affirmed the robustness of the coefficient estimates.

**Table 6:** Stepwise regression analysis (Global Quality Score).

Model	Predictors included	*R* ^2^	Adjusted *R*^2^	SE of the estimate	*F* change	*P* value	ANOVA	
							*F* test(df)	*P* value
1	Constant and PEMAT-U^a^	0.31	0.307	0.677	133.674	<.001	133.674 (1,298)	<.001
2	Constant, PEMAT-U, and PEMAT-A^b^	0.356	0.352	0.655	21.472	<.001	82.165 (2,297)	<.001
3	Constant, PEMAT-U, PEMAT-A, and duration	0.389	0.383	0.639	15.833	<.001	62.790 (3,296)	<.001

^a^PEMAT-U: Patient Education Materials Assessment Tool-Understandability.

^b^PEMAT-A: Patient Education Materials Assessment Tool-Accountability.

**Table 7:** Stepwise regression coefficients, statistical significance, and collinearity assessment (Global Quality Score).

Model and predictors		Unstandardized coefficients		Standardized coefficients	*t* test (df)	*P* value	Collinearity statistics	
		B (95% CI)	SE	β			Tolerance	VIF^a^
**Model 1**								
	(Constant)	−0.743 (−1.365 to −0.122)	0.316	—^b^	−2.354 (298)	0.019	—	—
	PEMAT-U^c^	0.051 (0.043 to 0.060)	0.004	0.556	11.562 (298)	<.001	1.000	1.000
**Model 2**								
	(Constant)	−0.800 (−1.401 to −0.198)	0.306	—	−2.615 (297)	0.009	—	—
	PEMAT-U	0.049 (0.040 to 0.057)	0.004	0.527	11.207 (297)	<.001	0.981	1.019
	PEMAT-A^d^	0.007 (0.004 to 0.009)	0.001	0.218	4.634 (297)	<.001	0.981	1.019
**Model 3**								
	(Constant)	−0.690 (−1.279 to −0.100)	0.300	—	−2.302 (296)	0.022	—	—
	PEMAT-U	0.045 (0.036 to 0.053)	0.004	0.485	10.317 (296)	<.001	0.933	1.072
	PEMAT-A	0.007 (0.004 to 0.010)	0.001	0.232	5.040 (296)	<.001	0.976	1.025
	Duration	0.001 (0.000 to 0.001)	0.000	0.186	3.979 (296)	<.001	0.949	1.054

^a^VIF: variance inflation factor.

^b^Not available.

^c^PEMAT-U: Patient Education Materials Assessment Tool-Understandability.

^d^PEMAT-A: Patient Education Materials Assessment Tool-Accountability.

**Table 8:** Stepwise regression analysis (modified DISCERN).

Model	Predictors included	*R* ^2^	Adjusted *R*^2^	SE of the estimate	*F* change	*P* value	ANOVA	
							*F* test (df)	*P* value
1	Constant and PEMAT-U^a^	0.15	0.147	0.525	52.66	<.001	52.66 (1,298)	<.001
2	Constant, PEMAT-U, and duration	0.202	0.196	0.509	19.147	<.001	37.507 (2,297)	<.001
3	Constant, PEMAT-U, duration, and PEMAT-A^b^	0.217	0.209	0.505	5.827	0.016	27.353 (3,296)	<.001

^a^PEMAT-U: Patient Education Materials Assessment Tool-Understandability.

^b^PEMAT-A: Patient Education Materials Assessment Tool-Accountability.

**Table 9:** Stepwise regression coefficients, statistical significance, and collinearity assessment (modified DISCERN).

Model and predictors		Unstandardized coefficients		Standardized coefficients	*t* test (df)	*P* value	Collinearity statistics	
		B (95% CI)	SE	β			Tolerance	VIF^a^
**Model 1**								
	(Constant)	0.550 (0.068-1.032)	0.245	—^b^	2.246 (298)	.025	—	—
	PEMAT-U^c^	0.025 (0.018-0.032)	0.003	0.388	7.257 (298)	<.001	1.000	1.000
**Model 2**								
	(Constant)	0.649 (0.179-1.119)	0.239	—	2.718 (297)	.007	—	—
	PEMAT-U	0.022 (0.015-0.029)	0.003	0.338	6.371 (297)	<.001	0.955	1.048
	Duration	0.001 (0.000-0.001)	0.000	0.232	4.376 (297)	<.001	0.955	1.048
**Model 3**								
	(Constant)	0.631 (0.164-1.097)	0.237	—	2.661 (296)	.008	—	—
	PEMAT-U	0.021 (0.014-0.027)	0.003	0.319	5.988 (296)	<.001	0.933	1.072
	Duration	0.001 (0.000-0.001)	0.000	0.242	4.584 (296)	<.001	0.949	1.054
	PEMAT-A^d^	0.003 (0.000-0.005)	0.001	0.126	2.414 (296)	.016	0.976	1.025

^a^VIF: variance inflation factor.

^b^Not available.

^c^PEMAT-U: Patient Education Materials Assessment Tool-Understandability.

^d^PEMAT-A: Patient Education Materials Assessment Tool-Accountability.

### Topic Modeling and Sentiment Analysis

Validation on a separate dataset confirmed the stability and efficacy of the sentiment analysis model used. Robustness checks, involving 10 independent training and evaluation runs initiated with distinct random seeds, produced mean performance metrics indicative of strong reliability: a mean accuracy of 0.8070 (SD 0.0113) and a mean weighted *F*_1_-score of 0.8017 (SD 0.0112) were achieved for the classification task. When deployed on the primary corpus (approximately 49,000 comments), the model classified most comments as neutral (35372/49680, 71.2%). Negative comments formed the next largest group (9141/49680, 18.4%), while positive comments were the least common category (5167/49680, 10.4%).

Application of the BERTopic algorithm to the comment corpus yielded 29 interpretable thematic clusters related to public perceptions and understanding of adolescent sexuality education, covering 87.3% (43371/49680) of the analyzed text. An additional, residual 12.7% (6309/49680) of comments fell into a miscellaneous category generated by the underlying bidirectional encoder representations from transformers (natural language processing) model for unclassifiable data, which was excluded from subsequent thematic analysis. These 29 meaningful topics were subsequently assigned to 6 overarching themes (Table 10). Examination of the prevalence associated with these topics offers direct insight into the subjects commanding the greatest public attention or dialogue. For instance, the considerable combined presence of topics within theme 3 (sources of knowledge acquisition) clearly signaled substantial public discussion and interest surrounding the channels—such as school, family, and online platforms—through which sexuality education is, or should be, delivered. Similarly, theme 2 (sexual safety education) emerged as significant, driven partly by the high individual prevalence of topic 0 (6857/43371, 15.8%), which focused on assault prevention and communication, thereby underscoring the public’s strong concern with safety and risk avoidance strategies.

**Table 10:** Themes related to public perceptions of adolescent sex education, along with corresponding topics. (N=43,371).

Theme and topic			Keywords (top 10)		Comments, n (%)
**Theme 1: adolescent physical development and physiological changes awareness**					
	Topic 3	menstruation, toilet, sanitary napkin, underwear, condom, clean, bathing, urethra, aunt^a^, vagina		3018 (7.0)	
	Topic 4	cm, height, average, 18cm, 15cm, 5cm, per capita, length, gōngfēn^b^, 14cm		2043 (4.7)	
	Topic 7	foreskin, retract, operation, to retract and expose, sensitive, expose, to have surgery, erection, the third kind, the second kind		1027 (2.4)	
	Topic 15	length, erection hardness, how long, size, dd^c^, skill, sexual endurance, measure, backstab, length and girth		977 (2.2)	
**Theme 2: sexual safety education—contraception, sexual harassment prevention, and self-protection**					
	Topic 0	to resist, sexual assault, masculinity, lack, violence, rape, assault, victim, feminization, to talk about sex		6857 (15.8)	
	Topic 6	girl, elder sister, gal, little sister, a girl, měinǚ^d^, pretty girl, respect for women, little girl, cross-dressing		1951 (4.5)	
	Topic 24	baby, hug, understood, sweetie, child, let me see, to force, to feel distressed for, got it, stay happy every day		467 (1.1)	
	Topic 26	to do well, protect, stay safe, be sure to protect, university student, measure, how old, protection, little angel, life		481 (1.1)	
	Topic 27	to go back, driving, to return home, to be late, send, close the door, hurry up, good person, to walk up onto, return first		508 (1.2)	
**Theme 3: sources of sexual knowledge acquisition—school-based, family-based, and online information channels**					
	Topic 2	biology lesson, biology teacher, second year of junior high school, first year of junior high school, lecture, to attend class, homeroom teacher, separate, grade 6, course		3153 (7.3)	
	Topic 5	to send out, in the group, to send to, what’s up?, this book, classic, official, emmm^e^, account, author		2173 (5.0)	
	Topic 9	popular science video, play, great, serious, excellent, to like, video, platform, detailed, awesome		1803 (4.2)	
	Topic 11	report, little child, reported by parents, parents and children, series, children and parents, children, complaint, count on, not teach		1769 (4.1)	
	Topic 19	mobile phone, computer, assignment, confiscate, play games, grades, to play games, National College Entrance Examination, to be addicted to, my little brother		949 (2.2)	
	Topic 21	self-taught talent, self-study, to figure out by oneself, study casually, study hard, focus on studying, study well, allow me, untaught, to acquire knowledge		551 (1.3)	
	Topic 23	to scroll and find, I browse, scroll, front page, to scroll and find a video, explosion, reflect, can scroll, check in, without reason or cause		426 (1.0)	
**Theme 4: sexual health, masturbation practices (benefits and risks), and impacts of pornography exposure**					
	Topic 1	sexual abstinence, desire, one week, moderate, to give up, one month, week, spirit, three times, harm		3736 (8.6)	
	Topic 8	Black person, bring, introduce, foreigner, foreign country/countries, Africa, to enter a country, take action, contagious, student studying abroad		998 (2.3)	
	Topic 18	to watch a movie, not watch, love to watch, TL:DR^6^ (too long didn’t read), watch too much, careful, fortunately, these words, take a look at a~, plot		1028 (2.4)	
	Topic 25	examine, traditional Chinese medicine (TCM), expert, treatment, doctor, regulate, experienced TCM doctor, to see a doctor, go see (a doctor) at the hospital, take medicine		710 (1.6)	
**Theme 5: sexual orientation, gender identity, and respect for diversity**					
	Topic 13	elder brother, guys, gay male, master, cute, unbelievable, my elder brother, invincible, eldest brother, driver		1145 (2.6)	
	Topic 16	have a meal, abnormal, review, while, devil, energy, music, to be at work, strawberry, public		1176 (2.7)	
	Topic 17	Acquired Immunodeficiency Syndrome, AIDS, infect, hpv, virus, transmit, test, male homosexuality, route, 'hiv		879 (2)	
	Topic 20	homosexuality, same-sex, group, heterosexuality, sexual orientation, discrimination, legal, proportion, gay man, gene		963 (2.2)	
**Theme 6: common psychological stressors and emotional regulation in adolescence**					
	Topic 10	responsible, exactly, responsibility, I will be responsible, department, don’t believe, irresponsible, meet, believe men, don’t understand		833(1.9)	
	Topic 12	joke, to joke, story, profile picture, can’t understand, funny, to be funny, talented person, hee hee^g^, upward		1601 (3.7)	
	Topic 14	magician, to be in love, deity, something years old, twenty, Beijing, twenty-something years old, mǔtāi^h^, under, never held		1241 (2.9)	
	Topic 22	scary, terror, fear, whimper, really?, number, terrified, terrible, monster, unknown		544 (1.2)	
	Topic 28	love, Chinese, woc^i^, real-name, have had, always have, lovely, and, jiao, me		364 (0.8)	

^a^“姨妈” is a common colloquial term or euphemism for menstruation in mainland China, literally meaning “aunt.”

^b“^公分” (gōngfēn) is a common, slightly more colloquial term for cm in Chinese, equivalent to “cm.”

^c^“dd” is a common Chinese internet slang term or abbreviation, originating from “didi” (meaning “younger brother”), which is commonly used as a euphemism for penis.

^d^“měinǚ” is very commonly used in mainland China as a general term of address for young or middle-aged women, sometimes not necessarily referring to the person’s outstanding appearance.

^e^“Emmm” is not a standard Chinese word, but rather mimics the sound of thinking, hesitation, or a slight pause, commonly found in online chat and spoken conversation.

^f^TL:DR is internet slang, meaning “save [data] flow.”

^g“^Hee hee” is an onomatopoeia representing laughter, often carrying connotations of a chuckle, a silly giggle, or a slightly sly and mischievous undertone.

^h^The pinyin “mǔtāi” (for the characters 母胎, literally means “mother’s womb”) is often used in the internet slang phrase “mǔtāi solo” (母胎solo), meaning “(someone who has been) single since birth” or “has never dated.”

^i^“Woc” is Chinese internet slang, usually a euphemistic substitute or abbreviation for the expletive “wǒ cào” (我操), used to express surprise, shock, dissatisfaction, or sometimes admiration, similar to interjections in English.

## Discussion

### Principal Findings

#### Knowledge

The ITGSE framework posits that CSE focused on “knowledge” should use a spiral, developmental curriculum covering 8 core conceptual areas to ensure learners progressively build essential understanding. Ideally, high-quality online videos would emulate this educational strategy. However, persistent gaps in sexual knowledge within target groups are evident. For example, data reported for 2023 by the Centers for Disease Control and Prevention showed a significant, concurrent drop in condom use among American youth, even as sexual activity and pregnancy rates fell, suggesting possible deficits in practical knowledge or risk perception [31]. Content available through popular digital platforms often fails to address these gaps comprehensively, frequently prioritizing topics perceived as high engagement (such as birth control methods) at the expense of fundamental CSE elements, such as STI prevention or discussions surrounding consent. This selective coverage occurs despite significant global health challenges, illustrated by recent Joint United Nations Program on HIV/AIDS (2024) statistics indicating 9.3 million people with HIV (including 43% of affected children) were without necessary medical care [6]. However, online video resources relevant to adolescent sexuality seldom offer thorough coverage of HIV prevention, thereby failing to fully use their potential for crucial public health messaging and service linkage in this critical area.

#### Attitudes

The ITGSE framework conceptualizes the “attitudes” component of sexuality education as fundamentally embedded within principles of human rights and gender equity. Therefore, optimal educational materials should purposefully confront harmful societal conventions and aim to diminish stigmatization. However, in practice, much online video content deviates from this ideal, often perpetuating established stereotypes and failing to address the distinct circumstances and requirements of marginalized groups, such as young people who identify as lesbian, gay, bisexual, transgender, queer+ or those with disabilities. The importance of tackling such disparities is highlighted by empirical evidence; for example, US data indicate Black female adolescents face a gonorrhea risk 8.8 times higher than their White counterparts, and young women with disabilities encounter double the likelihood of sexual assault compared to peers without disabilities [33]. By largely overlooking or failing to engage with these deep-seated structural inequalities, the capacity of current video content to foster significant attitudinal shifts and contribute to social change is substantially limited.

#### Skills

The ITGSE framework identifies “skills” development—encompassing areas such as interpersonal dialogue, assertion of boundaries (refusal), and accessing health care—as a key CSE component. While online video formats readily lend themselves to illustrating concrete actions (eg, demonstrating correct condom use), their utility often diminishes when addressing more intricate abilities, such as negotiation tactics or the cultivation of critical thinking about media messages. A relevant illustration of potential deficits in skills or adoption involves long-acting reversible contraceptives, including options such as intrauterine devices. These methods, despite proven high effectiveness, experience low global uptake [8,34]. This underuse is particularly striking given the comparatively modest use rates reported for condoms (approximately 38%) and oral contraceptive pills (approximately 27%) among young people in lower-resource settings [8,34]. Such discrepancies highlight an opportunity for well-crafted video interventions. By using lucid, engaging, and easily digestible presentation styles, videos could potentially narrow the divide between awareness of highly effective contraceptive options and their actual adoption or consideration by adolescents.

A correlation between platform publisher verification protocols and content quality is apparent; verified creators (eg, doctors) are prevalent on Bilibili and TikTok but rare on Kwai, contributing to the lower quality and trustworthiness of Kwai. The variations noted in video source distributions plausibly stem, at a minimum, from differing approaches to publisher verification across the platforms. TikTok, for example, mandates stricter validation for health care practitioners and imposes rigorous controls over medical advertising and pharmaceutical marketing. Kwai, conversely, typically requires only fundamental real-name confirmation for its individual content posters. Intriguingly, Kwai registered the most substantial user interaction metrics, notwithstanding its inferior content quality. This discrepancy suggests the potent effect of market-oriented algorithms prioritizing engagement, a dynamic risking magnification of inaccurate or false information. Approximately one-tenth of the examined videos propagated factual errors or misleading statements (eg, asserting human papilloma virus does not affect males or that condoms fail to prevent HIV transmission). Such findings highlight a profound digital-age hazard—readily available, unmediated online content can expose adolescents to significantly harmful viewpoints.

One particularly notable outcome of this investigation was identifying institutional media as the origin for 5.7% (17/300) of the analyzed videos. We believe this finding to be unique, as previous studies focusing on health information dissemination for other conditions via video platforms have not typically reported such a presence. This observation lends itself to a 2-fold interpretation regarding the state of adolescent sexuality education in China. First, it could indicate a heightened focus by governmental bodies on this area as a key public health issue. Alternatively, the very necessity for media channel involvement, coupled with the relatively small percentage observed, could paradoxically highlight persistent shortcomings or voids within the established educational systems tasked with providing CSE.

Sexuality education requires careful instructional methods, focusing crucially on fostering attitude and perspective changes—often the most formidable barrier—beyond simply conveying information. Active parental engagement, including self-education and age-calibrated strategies, is also vital for an optimal learning environment. Internationally, China’s provision significantly lags behind many Western nations, such as France, with its notably thorough curriculum [35]. Our analysis found that approximately 30% of the reviewed videos alluded to these shortcomings within the Chinese context. This reflects awareness that prevalent societal taboos (“tán sè xìng biàn”) discourage open conversation, engendering widespread ignorance, reducing risk management abilities, and ultimately leading to avoidable negative consequences.

Studies confirm that while comprehensive and web-based sexuality education can effectively lower teenage birth rates, they typically exert only a marginal influence on altering actual sexual practices [11,36]. A significant complication for effective delivery is the notably low participation of health care providers, who are ideally positioned as sexual health advocates. This study highlights this specific gap, identifying a strikingly small percentage of physicians among video creators—a finding inconsistent with patterns noted in other scholarly work [23,35,37]. Supporting this, the 2016 clinical report by the American Academy of Pediatrics acknowledged the limited involvement of pediatricians in this domain. [38].

The low engagement of physicians on internet platforms is concerning, as it not only reflects a deficiency in the supply of educational content but also potentially exacerbates the knowledge disparity among adolescents in accessing accurate, professional sexual health information. This disparity may be influenced by socioeconomic or geographic factors, such as economic or regional differences in how frequently urban and rural children visit physicians, directly affecting their opportunities to obtain sexual health knowledge through traditional channels. If physicians, as sexual health advocates, fail to fully leverage internet platforms, adolescents who rely on online resources for information, especially those in medically underserved areas, may be more susceptible to inaccurate or incomplete sex education content, thereby widening health information inequities.

Future efforts should strive to rectify this imbalance, focusing on first, fostering unified home and school sexuality education strategies to ensure the dissemination of foundational knowledge, and second, and more critically, actively exploring how social media platforms can help correct this disparity. This could involve platform mechanisms that encourage more physicians to publish high-quality sexual health content and grant it greater visibility. By integrating physicians more deeply into educational settings (eg, “physicians-in-schools” programs or online expert lectures), young people’s understanding of sexuality and reproductive well-being can be significantly bolstered.

### Correlation and Stepwise Regression Analysis of Video Quality, Reliability, and Video Parameters

Correlation analysis revealed that a higher number of followers generally correlated with increased comments, shares, and likes, which aligned with social media platform dissemination patterns. A moderate positive correlation (*r*=0.46) was observed between GQS and mDISCERN, suggesting that user-perceived quality and reliability change in parallel to some extent, though they are distinct concepts. Video duration also showed some association with information disclosure and quality scores. Longer videos may contain more information (higher mDISCERN), thus leading to higher-quality ratings (GQS). It is possible for a well-produced and easily understandable video (high GQS) to be deemed less reliable (low mDISCERN) due to an unclear source or lack of evidence. Understandability (PEMAT-U) serves as a crucial link, vital for enhancing both. A critical takeaway is the substantial potential for enhancing actionability across all identified content domains. Consequently, content developers are advised to focus on improving the incorporation of practical, actionable steps within knowledge-centric videos—most notably for subjects such as preventing sexual assault, contraception use, and STD and AIDS prevention—aiming to elevate both the user experience and the overall educational outcomes.

In concert, understandability (PEMAT-U), actionability (PEMAT-A), and duration were identified as significant factors predicting video quality (GQS), with understandability demonstrating the most substantial influence. While these same 3 variables also held predictive value for video reliability (mDISCERN), the model’s overall capacity to explain variance was weaker in this case, and the specific impact of actionability was marginal (*P*=.02). The stepwise regression technique used offers benefits, such as automated variable selection, ease of use, suitability for initial exploration, and the possibility of achieving model parsimony. Nevertheless, despite its frequent application, this methodology possesses inherent constraints. A key limitation is its tendency to yield a model that is statistically optimal for the given dataset (“locally optimal”) but may not align with the most theoretically sound or practically relevant model. Therefore, interpreting and extending the results derived from the final regression models generated via this approach warrants a degree of prudence.

### Topic Modeling and Sentiment Analysis

Sentiment analysis of user comments revealed that discussions surrounding the sexuality education videos were generally neutral, mainly focusing on nonemotional factual exchange or discussion about the educational process, possibly also including user uncertainty or contradictory feelings. Notably, negative comments (9141/49,680, 18.4%) significantly outnumbered positive comments (5167/49,680, 10.4%). This may reflect users’ concerns, dissatisfaction, or criticism regarding sensitive topics (such as safety, risks, prevention, etc), information quality, or personal experiences. Overall, the sentiment landscape highlights the challenges and potential anxieties faced by adolescent sexuality education in the digital space.

Topic modeling of over 43,000 comments revealed the aspects that most resonated with the audience. Users highly focused on the sources and methods of obtaining sexuality education (theme 3), and strongly focused on sexual safety and prevention (theme 2, especially sexual assault prevention [topic 0]). Discussions about physical development (theme 1) and sexual practices and health (theme 4, including masturbation and pornography impact) were also prominent, suggesting that these areas may lack sufficient information or be controversial. Although the proportion was lower, diversity and identity (theme 5) and psychological stress (theme 6) were also part of the discussion. The overlap between themes indicates that these topics are interconnected in public discussion.

### Linking Engagement and Feedback

While the present regression models did not specifically test correlations between topic frequency and platform-level user interaction, broader results suggest that content quality and reliability may not align with audience engagement. Substantial interaction on platforms such as Kwai, often from lower-quality creators, may stem from a focus on provocative or less moderated themes (eg, physical development, sexual behavior, etc). Conversely, higher quality, reliable content from verified sources addresses more subtle CSE dimensions (eg, consent, diversity, and prevention). Consequently, their nuance and depth may result in lower mass interaction on platforms where algorithms prioritize engagement over content attributes.

### Strengths and Limitations

This study analyzed adolescent sexuality education videos on major Chinese short-video platforms. Assessment by experts in medicine and education used GQS, mDISCERN, and PEMAT tools, focusing on video quality, reliability, clarity, and actionability. The study also innovatively integrated sentiment analysis and topic modeling of user comments to gauge audience reception and experience. Findings yield actionable recommendations for improving video content quality.

However, this study faces several limitations. First, the exclusive focus on Chinese-language content inherently limits the cross-cultural generalizability of our findings. Second, analyzing only the top 100 videos may introduce platform algorithm bias. Third, the applicability and validity of GQS and mDISCERN for this video genre require further validation. Finally, videos from institutional media and professional educators were underrepresented in the sample. Future research should address these limitations.

Despite screening the top 100 videos by comprehensive ranking to identify mainstream content, it is crucial to acknowledge the potential influence of platform algorithmic characteristics on video visibility and ranking. Douyin’s algorithm, known for favoring short, visually impactful content, likely prioritizes videos aligning with these features, potentially leading to an underrepresentation of longer, more in-depth discussions. The algorithm by Kuaishou, while also emphasizing short-form video, incorporates a strong social-gifting and localized community focus, which can amplify content that drives immediate user interaction and caters to specific community preferences. In contrast, the algorithm by Bilibili prioritizes community engagement and is more compatible with medium-to-long-form content, potentially yielding a different video distribution.

This inherent algorithmic preference or bias can impact the generalizability of our findings regarding CSE information across the entire platform ecosystem and may introduce a representational bias in the analyzed content. The phenomenon of “high engagement, low quality” is often exacerbated by these mechanisms. Algorithms optimize for metrics such as views, likes, and shares, which do not inherently correlate with factual accuracy or educational quality. This can lead to a feedback loop where engaging but potentially misleading content gains prominence. Future research should adopt differentiated sampling strategies that account for these varying algorithmic behaviors to more comprehensively capture the online adolescent sexual health information landscape.

### Suggestions

This research provides valuable insights for enhancing online adolescent sexuality education videos. Drawing lessons from the success of evidence-based frameworks such as ITGSE [6], key recommendations emphasize improving content clarity (PEMAT-U) and actionability (PEMAT-A) through concrete guidance and building reliability via transparent sourcing, evidence citation, and neutrality. Incorporating diverse voices (eg, youth with disabilities and sexual or gender minorities) and considering appropriate video duration are vital for inclusivity and comprehensive information delivery. Video platforms should collaborate with authoritative institutions and experts to strengthen content and publisher verification and optimize recommendation algorithms to prioritize evidence-based, high-quality materials over simple engagement metrics, potentially offering credibility assessment tools. Fostering media and information literacy among educators and users is crucial for critical evaluation of online information and safeguarding health. Future research should further explore strategies for evaluating and improving the digital sexuality education ecosystem.

### Conclusions

This investigation assessed the informational quality of adolescent sexuality education videos on China’s Bilibili, TikTok, and Kwai platforms. Key findings showed quality disparities. Bilibili and TikTok offered moderate-quality content, while Kwai had typically lower-quality content. Notably, videos from authenticated physicians and educators consistently achieved higher quality and reliability. This reflects a growing disparity in platform quality standards and professionalism. Entertainment-focused platforms such as Kwai are less advisable for reliable educational content compared to platforms such as Bilibili, which feature more academic material requiring critical user evaluation.

Directions for future research include exploring methods to improve platform-specific quality benchmarks and enhancing viewer media literacy for online health information. A challenge remains in reconciling professional credibility with broad appeal and accessibility. Video platforms hold significant, often unrealized, potential to transform adolescent sexuality education by featuring inclusive, skills-focused, evidence-based content.

Despite existing deficiencies, aligning with established guidelines, such as ITGSE, offers a clear path for advancement. These efforts can empower youth through quality education, enabling platforms to contribute to a healthier and more equitable future for Chinese adolescents, particularly by addressing goals related to STI education, pregnancy prevention, and fostering a more open societal attitude toward sexuality in China.

## Appendix

Multimedia Appendix 1 Dataset on adolescent sex education.

Multimedia Appendix 2 Quality assessment tools.

## Abbreviations

CSE: comprehensive sexuality education
GQS: Global Quality Score
ITGSE: International Technical Guidance on Sexuality Education
mDISCERN: modified DISCERN
PEMAT: Patient Education Materials Assessment Tool
SDG: sustainable development goal
STD: sexually transmitted disease
STI: sexually transmitted infection

## Data Availability

All data generated or analyzed during this study are included in this published paper and its multimedia appendices. The raw video and comment data analyzed are publicly available on the Bilibili, Douyin (TikTok), and Kwai platforms, subject to their terms of service and content availability.
