# Supplementary material for: Quality and Reliability of Adolescent Sexuality Education on Chinese Video Platforms: Sentiment-Topic Analysis and Cross-Sectional Study
Source: JMIR Form Res. 2025 Sep 5;9:e77100. doi: 10.2196/77100 (PMC12449667; doi:10.2196/77100)
Supplement: Multimedia Appendix 2 [file formative_v9i1e77100_app2.docx]

**Quality Assessment Tools**

**Description of the global quality score(5-point scale)for evaluating the quality of the videos.**

| Score | Description |
| --- | --- |
| 1 | Poor quality,poor flow of the site,most information missing,and it is useless for patients. |
| 2 | Generally poor quality and flow,the content logic is poor, although some information is listed, more important information is still missing, and the use of patients is very limited. |
| 3 | Moderate quality,suboptimal flow,some important information is adequately discussed, somewhat useful for patients. |
| 4 | Good quality and flow,the video logic is clear and smooth,most of the relevant information is listed,useful for patients. |
| 5 | Excellent quality and flow,the video logic is clear, and the content is very smooth, very useful for patients. |

**Modified DISCERN quality criteria for assessing the reliability of video. (1 point for answer ‘yes’, 0 point for answer ‘no’)**

| **Reliability Score** |
| --- |
| 1. Is the video clear, concise, and understandable? |
| 2. Are valid sources cited? |
| 3. Is the content presented balanced and unbiased? |
| 4. Are additional sources of content listed for patient reference? |
| 5. Are areas of uncertainty mentioned? |

1 = Poor (Very low reliability)
2 = Fair (Limited reliability)
3 = Moderate (Adequate reliability)
4 = Good (Substantial reliability)
5 = Excellent (High reliability)

JH and WY independently reviewed and scored the videos. Any discrepancies in GQS and mDISCERN ratings between the two reviewers were adjudicated by XS to determine the final scores.
